# Discovering semantic features in the literature: a foundation for building functional associations

**DOI:** 10.1186/1471-2105-7-41

**Published:** 2006-01-26

**Authors:** Monica Chagoyen, Pedro Carmona-Saez, Hagit Shatkay, Jose M Carazo, Alberto Pascual-Montano

**Affiliations:** 1 Biocomputing Unit, Centro Nacional de Biotecnologia – CSIC, Madrid, Spain; 2 School of Computing, Queen's University, Kingston, Ontario, Canada; 3 Dpto. Arquitectura de Computadores, Universidad Complutense de Madrid, Madrid, Spain

## Abstract

**Background:**

Experimental techniques such as DNA microarray, serial analysis of gene expression (SAGE) and mass spectrometry proteomics, among others, are generating large amounts of data related to genes and proteins at different levels. As in any other experimental approach, it is necessary to analyze these data in the context of previously known information about the biological entities under study. The literature is a particularly valuable source of information for experiment validation and interpretation. Therefore, the development of automated text mining tools to assist in such interpretation is one of the main challenges in current bioinformatics research.

**Results:**

We present a method to create literature profiles for large sets of genes or proteins based on common semantic features extracted from a corpus of relevant documents. These profiles can be used to establish pair-wise similarities among genes, utilized in gene/protein classification or can be even combined with experimental measurements. Semantic features can be used by researchers to facilitate the understanding of the commonalities indicated by experimental results. Our approach is based on *non-negative matrix factorization *(NMF), a machine-learning algorithm for data analysis, capable of identifying local patterns that characterize a subset of the data. The literature is thus used to establish putative relationships among subsets of genes or proteins and to provide coherent justification for this clustering into subsets. We demonstrate the utility of the method by applying it to two independent and vastly different sets of genes.

**Conclusion:**

The presented method can create literature profiles from documents relevant to sets of genes. The representation of genes as additive linear combinations of semantic features allows for the exploration of functional associations as well as for clustering, suggesting a valuable methodology for the validation and interpretation of high-throughput experimental data.

## Background

Experimental techniques such as DNA microarray, serial analysis of gene expression (SAGE) and mass spectrometry proteomics, among others, have opened new ways to study biological systems from a global perspective. These new methodologies are generating large amounts of data related to genes and proteins at different levels. As in any other experimental approach, it is necessary to analyze these data in the context of the previously known information about the biological entities under study. However, obtaining and interpreting biological knowledge from these large sets of data is not a trivial task. Consequently, the development of automatic methods to assist in functional interpretation is one of the main challenges in bioinformatics research.

The advent of on-line access to biomedical literature in the last few years has generated a broad interest in text analysis tools for automatic extraction of latent information and knowledge about almost any topic in science. Since biomedical literature covers all aspects of biology, chemistry, and medicine, there is almost no limit to the types of information that may be recovered through careful and exhaustive mining [1]. Therefore, the literature is a valuable source of information to be automatically analyzed for experiment validation and interpretation.

Furthermore, database curators make extensively use of the biomedical literature in order to find evidence supporting functional information of genes and proteins. This information is ultimately available as annotations using controlled vocabularies, ontologies, free-text and reference to relevant publications. Although text analysis can support database curation and experimental interpretation, these two applications are distinct areas, as they address different requirements and different problems. On one hand, methods and tools for supporting experts in database annotation work with very large document collections (in the extreme case a whole bibliographic database such as MEDLINE, or typically, an organism-specific subset of MEDLINE [2]). In addition, such applications have stringent search criteria which enable the definition of a relevance metric (e.g., the search for articles describing proteins associated with biological processes). Due to these characteristics, such methods are typically part of the broad category of *information retrieval *although in some cases they also require an *information extraction *component to find explicit entities and facts within the unstructured text. On the other hand, methods developed to assist researchers in the interpretation of genome-wide experiments can be applied to much smaller literature collections (in some cases, only the known relevant articles associated with the gene/protein), and the precise information to be extracted is generally not well-defined. Therefore, these methods can be generally described as *text mining *[3]. Undoubtedly, the latter can benefit from bibliographic annotations available in curated databases. Likewise, literature annotations will not be used to their full extent by bench scientists if they cannot rely on automatic tools for their analysis.

One of the necessary steps in supporting high-throughput genomic and proteomic experiments is the attribution of functional meaning to the results (e.g. the set of genes differentially expressed obtained in a DNA microarray experiment or a list of protein identifications obtained by mass spectrometry). During the last few years, several literature analysis methods have been proposed to support such functional analysis of genes and proteins. Co-occurrence based methods associate genes and proteins if their names co-occur within some scope of the literature (abstract, document, paragraph or sentence). Such methods use gene nomenclature [4], thesaurus concepts [5], or generalized objects (namely gene names, diseases, phenotypes and chemical compounds) [6]. These methods usually need large literature collections as well as powerful methods for entity/name detection. Therefore, co-occurrence methods typically rely heavily on information extraction techniques. Other methods identify functional relations among pre-clustered genes by inferring terms that are significantly associated with gene expression clusters [7], or alternatively, by using clusters that exhibit similarity in both expression and related literature [8], or by calculating a score that indicates text-based functional coherence [9]. While these methods are useful in some experimental environments, they are of limited use when the experimental techniques do not readily produce gene/protein subsets (e.g. protein identifications obtained by mass spectrometry).

Finally, a number of text mining methods rely on document similarity measurements (originally developed in the context of information retrieval) to establish relationships among genes based on their associated literature. Shatkay *et al*. [10] used a probabilistic model [11] to suggest gene relationships and provide a keyword list associated with each gene. Chaussabel and Sher [12] performed a two-way hierarchical clustering of documents represented by term-frequency vectors [13,14] to find gene relationships as well as local patterns of terms associated with gene subsets. Glenisson *et al*. [15] investigated value of the vector space model for gene clustering, based on a divisive clustering algorithm. (Several other groups have used the vector space model to represent and cluster documents in a variety of biomedical applications [16-18]). More recently, Homayouni *et al*. [19] used the latent semantic space obtained by means of Singular Value Decomposition (SVD) [20] to group genes, applying hierarchical clustering. The benefit of these methods is that they provide a literature profile (by means of different document representations) for each gene/protein of interest. These profiles are then used to perform further analysis like pair-wise comparisons, clustering or are even combined with experimental measurements [21]. Clearly, there is still much room for improvement on all these methods, and biomedical text mining is a widely open research area.

In this paper we propose a novel application of a different data analysis method, *non-negative matrix factorization (NMF)*, to create literature profiles and establish relationships within large sets of genes/proteins from a corpus of documents known to be relevant to each gene/protein. NMF was introduced a few years ago [22] in another computational context, (originally applied to image as well as to text analysis), and more recently it has been used to analyze gene expression [23,24], sequence data [25], and gene functional annotations [26].

The document representation obtained by NMF provides advantages over previous representations as it combines the best properties found in other models [27-29]. First, like other factorization methods, it reduces the dimensionality of the initial space originally formed by terms and documents. Second, the new basis vectors, produced by the factorization, provide a succinct list of positively weighted terms that can be interpreted as "executive summaries", while providing a new representation of documents, as additive combinations of the basis vectors. In contrast, the classical singular value decomposition (SVD), which combines positive and negative mixtures, produces features which lack intuitive meaning [22].

Briefly, key steps in the proposed methodology are as follows. First, for each gene in the data set we create a broad document (which we call the *gene-document*). It is produced by concatenating all the relevant abstracts and titles associated with the gene in the corpus. Second, each gene-document is converted into its vector space representation. Third, by applying NMF to the vector representation, we transform each gene into a literature profile that captures its relative relevance in a new set of basis vectors. Lee and Seung [22] used the term *semantic features *to refer to the basis vectors discovered by NMF, since these vectors consist of a weighted list of terms that are semantically related. In this work we have examined the semantic features obtained by NMF to assess their potential value as functional descriptors. Finally, semantic profiles are further used to analyze relationships among genes, as well as to classify genes into coherent functional groups.

In this way, the literature is used to first, discover the main semantic features associated with a large list of genes, and second, to establish putative relationships among subsets of genes while providing a sound justification for this classification. The information obtained by our method can be further used to interpret and validate high-throughput experimental results. For simplicity and clarity of the exposition we only refer to genes, although the methodology is applicable to genes, proteins, and potentially other types of entities discussed in the biomedical literature. It is important to mention that the proposed method requires a collection of literature references that are relevant to each gene (here referred to as the *literature corpus*).

In summary, our method is able to:

• Discover *semantic features *from a literature corpus generated for a set of genes, providing a semantic profile for each gene.

• Suggest functional associations among genes based on their similarity in the discovered semantic space.

To evaluate our method, we used two gene datasets. The first, created for this purpose, contains 575 *Saccharomyces cerevisiae *genes associated with eight broad biological processes. The second contains 50 genes related to cancer biology, Alzheimer's disease and development (referred to as the Reelin dataset). This set was proposed and analyzed by Homayouni et al. (2005) [19] to identify conceptual gene relationships. To build the corresponding literature corpora, we used two databases containing bibliographic references annotated by experts. In the case of the yeast set, literature references were obtained from the *Saccharomyces *Genome Database (SGD) [30] while in the case of the Reelin dataset, references were obtained from Entrez Gene [31,32]. We provide both datasets and corresponding literature corpora as supplementary data in the project web page to support future comparative studies [33]. The selection of curated bibliographic references ensures that the texts to be mined are relevant to the genes in each set.

## Results

### General schema of the method

**Figure 1:**
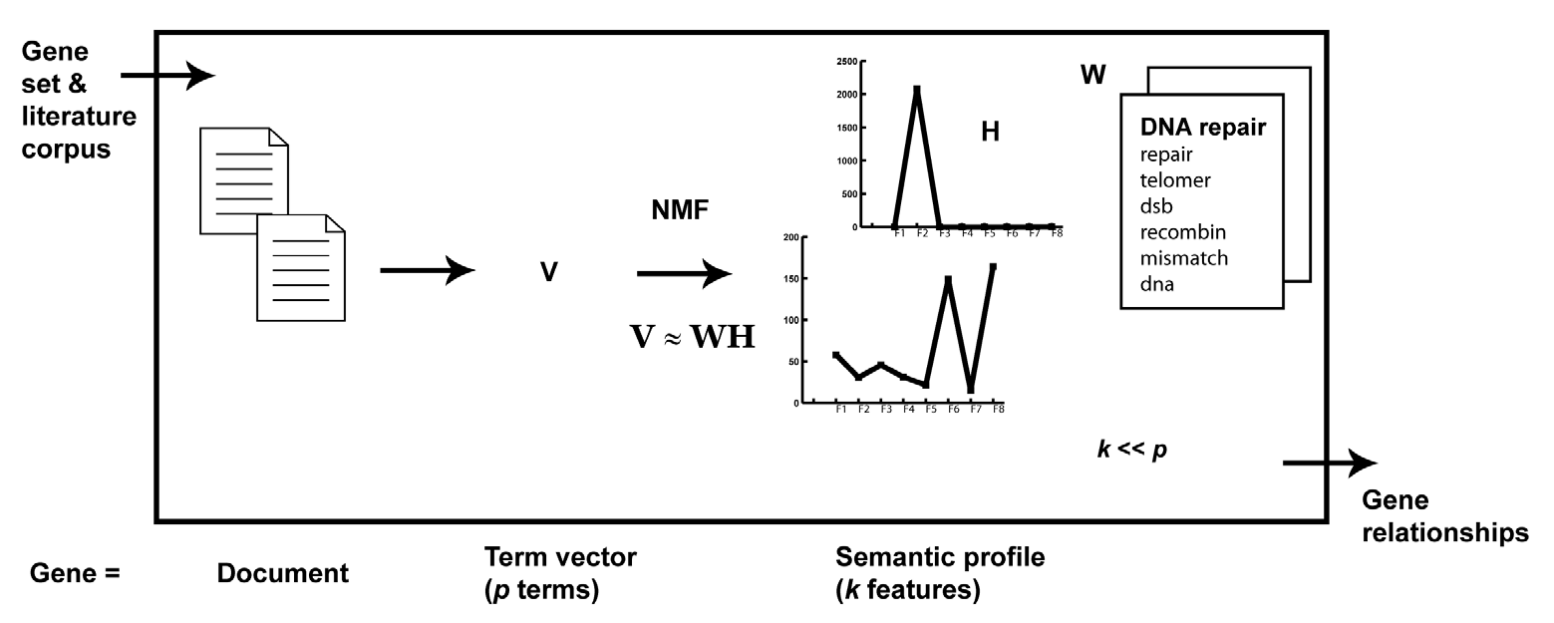
**Method overview**. Schematic overview of the method and corresponding gene representation.

Briefly, our method proceeds as follows (see Figure 1): a broad gene-document is constructed for each gene by concatenating its relevant bibliographic references (abstracts and titles). A vector space representation, namely, a weighted term-frequency vector, is built for each gene-document. This term-based space (**V**) is mapped by means of Non-negative Matrix Factorization (NMF), to a lower-dimensional representation based on semantics features (**W**), obtaining a new semantic-based space, where genes are represented through semantic profiles (**H)**. Gene relationships are established by cluster analysis of the gene semantic profiles. A detailed description of the methodology is provided in the Methods section.

### *S. cerevisiae *dataset

To assess the performance of our methodology we used a set of *Saccharomyces cerevisiae *genes for which functional annotations are well-established, providing a basis for assessing the value of our results. To construct this set we selected eight biological process categories from the SGD Gene Ontology Slim Mapper [34]. As GO Slim Mapper provides expert annotation of SGD genes to a set of high level GO categories, we can use these annotations as a gold standard to compare our results against. The chosen categories are: 'cell cycle', 'cell wall organization and biogenesis', 'DNA metabolism', 'lipid metabolism', 'protein biosynthesis', 'response to stress', 'signal transduction' and 'transport' (see table 1).

**Table 1:** Biological processes in test data set (according to GO Slim annotations in "Biological Process Category")

**GO Code**	**GO Name**	**Number of genes**	**Common genes**
GO:0007049	cell cycle	77	5 DNA metabolism
			2 response to stress
GO:0007047	cell wall organization and biogenesis	32	3 signal transduction
GO:0006259	DNA metabolism	146	5 cell cycle
			1 transport
GO:0006629	lipid metabolism	34	1 response to stress
GO:0042158	protein biosynthesis	49	-
GO:0006950	response to stress	63	2 cell cycle
			1 signal transduction
			4 transport
			1 lipid metabolism
GO:0007165	signal transduction	39	3 cell wall organization and biogenesis
			1 response to stress
GO:0006810	transport	152	1 DNA metabolism
			4 response to stress

Genes annotated with any of these eight categories were obtained from the SGD database [30]. The gene set was further filtered so as to retain those genes having a similar number of annotated references. Thus, the chosen genes all had between 10 and 100 bibliographic references under the "Function/Process" annotation category. This selection resulted in the final set of 575 genes (referred to hereafter as the SGD8 dataset), with a literature corpus comprising a total of 7,080 distinct articles. Therefore, this dataset contains a heterogeneous set of genes related to different expert-annotated biological processes, which are expected to be covered in the associated literature. This data is a valuable test set to assess our method, as well as for assessing the value of the semantic features for functional interpretation, since we examine here whether the gene relationships and the semantic features – automatically extracted by NMF – agree with the relationships that can be established by curated annotations.

We then constructed a term-frequency vector representation from the literature associated with each gene. Terms were filtered out if they did not appear in at least 4% of the genes, or alternatively, if they appeared in more than 80% of the genes. These cutoff values were chosen as they have proven good in practice, to remove very frequent terms which are not good discriminators among genes and, at the same time, to remove very rare terms which are only relevant to a few genes in the set and are therefore not good characteristics for relationships among them [14]. The resulting gene-term matrix after this process contains 575 vectors (genes) and 2,365 variables (terms). In practice, cutoff values should be established within reasonable limits to obtain robust results. Our experiments with the SGD8 dataset suggest that terms can be filtered if found in less than 28 genes (as uncommon terms will be filtered in any case in the factorization step) and the cutoff filter for common terms should not be less that 60% (to ensure that semantic features contain general biological terminology that support easy interpretation).

The selection of the number of factors *k *(or semantic features) for this data set was done using the model selection method proposed by Brunet *at al. *(2004) [24]. Briefly, the cophenetic correlation coefficient is used as a measure of the robustness of the method in producing stable groups of genes from different random initializations for a given number of factors (*k*). Based on this estimation, a value of *k *= 8 was selected from a set of 100 independent runs, (with *k*'s value ranging from 2 to 16, see Additional file 1). Usually the value of *k *is selected at the point where the magnitude of the cophenetic correlation coefficient shows a significant expression in the form of a peak. However, it is possible that several peaks appear for different values of *k*, which indicates that there are multiple possible stable solutions. In general, higher values of *k *will reveal more localized and specific semantic features in the literature. In our case we selected the value *k *= 8 because it represented the minimum number of recognized stable features, although more detailed features could also be found if a higher *k *value was chosen.

### Analysis of semantic features

The rational behind our approach lies in the ability of NMF to transform the representation of gene-documents from a high-dimensional vector of term counts into a lower-dimensional, additive linear combination of semantic features. Both the features and the linear combination are simultaneously inferred during an iterative learning process. Unlike other matrix decomposition methods, e.g. latent semantics indexing via SVD, NMF enforces a linear combination using only nonnegative coefficients, and therefore, unlike SVD, NMF tends to produce a decomposition of the data under analysis into readily-understood components [22]. In the context of text analysis, this decomposition produces features, which are essentially *sets of terms *as found in a subset of the original data. Note that the widely-used SVD does not produce such terms, but rather abstract weight combinations that are not easily interpreted. The set of terms produced by NMF were referred to as *semantic features *by Lee and Seung (1999) as they are usually topically related.

We have examined the semantic features obtained by NMF to assess whether the relatedness of the terms within semantic features supports their use as biological descriptors. Each semantic feature consists of an ordered list of terms, sorted by their respective weight in the **W **coefficient matrix, where only a few of the terms that were originally extracted from the corpus have non-zero value. This way, the semantic features could help researchers in interpreting the obtained literature profiles by highlighting the most significant terms of each feature. Genes, which are in turn viewed as additive combinations of semantic features – called semantic profiles – can be characterized using the terms occurring within their respective features.

Although the terms included in each semantic feature appeared topically related to the authors we performed an additional unbiased assessment. To guarantee the independence and objectivity of the evaluation, four molecular biologists (including only one specialist in microbiology among them), were independently asked to interpret the top 10 terms in each semantic feature, providing a free text label that summarizes the biological topics that each term set suggested. To avoid bias in the interpretation, GO Slim categories were not provided to the experts. The experts were able to identify a coherent biological context for each feature represented by the top terms in the list. For seven of the eight features the four experts were in complete agreement, although labels vary slightly, as features were interpreted at several levels of abstraction (e.g. mitosis vs. cell division, vesicular trafficking vs. transport, DNA repair vs. DNA metabolism). Only one of the eight features (feature 3) was subject to three different interpretations, revealing a possible mixture of base topics (metabolism, stress response, protein degradation). Table 2 shows the semantic features (*k *= 8), discovered in a factorization experiment for the SGD8 dataset as an ordered list of top 10 terms, and the corresponding labels provided by experts.

**Table 2:** Example semantic features (SGD8 dataset). Top 10 terms in the k = 8 semantic features obtained for a NMF experiment (ordered by decreasing importance). Labels show topical interpretations provided by experts (including more concrete topics in parenthesis)

**F1 DNA metabolism (DNA replication)**	**F2 DNA metabolism (DNA repair)**	**F3 Metabolism/Stress/Degradation**	**F4 Transciption (chromatin)**	**F5 Cell Division (mitosis)**	**F6 Miochondria**	**F7 Transport (vesicular trafficking)**	**F8 Protein synthesis**
replic	repair	glucos	actin	spindl	mitochondri	transport	translat
pcna	telomer	fatti	swi	cyclin	preprotein	vesicl	mrna
dna	dsb	heat	nucleosom	kinetochor	mitochondria	vacuolar	trna
ner	recombin	stress	snf	hsp90	inner	vacuol	alpha
damag	mismatch	endoplasm	histon	chaperon	transloc	membran	gcn4
checkpoint	dna	reticulum	chromatin	scf	outer	nitrogen	beta
rfc	rad52	proteasom	elong	anaphas	membran	secretori	gtp
pol	excis	phosphatas	mate	mitosi	matrix	autophagi	phosphoryl
polymeras	rad51	atpas	silenc	centromer	oxid	cytoplasm	exchang
rad6	endonucleas	sphingolipid	polar	mitot	translocas	sort	kinas

**Figure 2:**
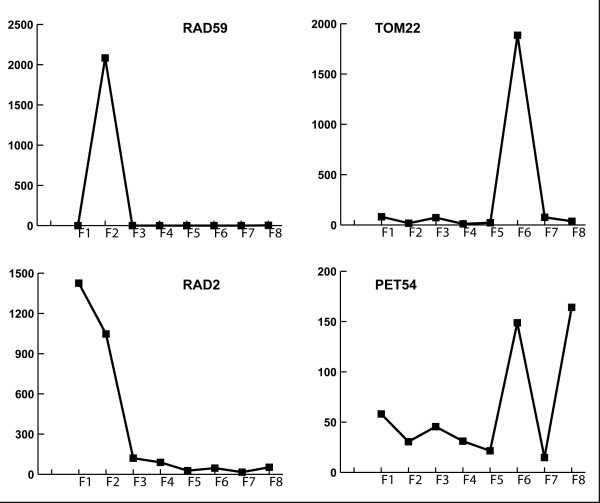
**Example semantic profiles**. Genes are represented as semantic profiles (linear combination of semantic features). Profiles of some of the genes in the SGD8 dataset are shown, using semantic features (F1 to F8) in table 2.

To summarize this stage, NMF provides a new representation scheme, mapping the initial high-dimensional gene-term representation, to an additive linear combination of features (gene semantic profile) in a lower-dimensional space. The semantic profile provides information about the relative weights of semantic features in the corpus relevant to a given gene (Figure 2). The latter representation is a good fit for the case of functional information as it takes into account the significant number of gene products that perform multiple functions in the cell. Also, several aspects of the functional characterization of a gene are described in the literature. In this way, some genes are associated mainly with one semantic feature. For instance, *RAD59*, involved in the repair of double-strand breaks in the DNA, has a profile that shows a clear component of feature 2 (labeled as *DNA repair *by the experts); *SNF2*, encodes the catalytic subunit of the SWI/SNF chromatin remodeling complex, has a high value at feature 4 (labeled as *chromatin*); *TOM22*, a constituent of the mitochondrial outer membrane translocase complex involved in protein import into mitochondria, is strongly associated with feature 6 (labeled as *mithochondria*); and *ATG9*, encodes a transmembrane protein involved in formation of Cvt and autophagic vesicles, is clearly associated with feature 7 (labeled as *transport*).

Other genes have multi-feature profiles, showing relatively high values for more than one feature. E.g. *RAD2 *(features 1 and 2, labeled as *DNA replication *and *DNA repair *respectively) is a single-stranded DNA endonuclease that cleaves single-stranded DNA during nucleotide excision repair to excise damaged DNA; and *PET54*, which encodes a protein required for splicing the COX1 intron AI5 beta, also specifically required together with Pet122p and Pet494p, for translation of the COX3 mRNA, is located in the mitochondrial inner membrane (in this case the highest weights are given to features 6 and 8, labeled as *mitochondria *and *protein synthesis *respectively).

To further verify that the interpretability of the semantic representation originates in the data, and is not an artifact of the proposed methodology we performed the same analysis using a random dataset. The initial data, i.e. gene-term frequency matrix corresponding to the SGD8 set, was randomly perturbed by iterative shuffling of elements in each row and column. Low cophenetic correlation coefficients (in the range of 0.4–0.5 for random data compared to ~0.95 values in SGD8 set) revealed the impossibility of finding stable groups along different factor values (see Additional file 1). In addition, the four experts could not find any common biological topic to the set of terms comprising each of the obtained features.

### Inferring functional relationships from semantic profiles

Our assumption, which is further justified by our results, is that semantic features discovered by NMF analysis of gene literature provide an interpretable, reduced dimensionality space in which functional relationships among genes can be established. That is, genes represented by similar semantic profiles are indeed functionally related.

In order to verify the value of the semantic profiles to establish similarities among genes we performed a clustering experiment to find functionally coherent groups. To provide a robust clustering, we exploit the non-deterministic nature of the NMF algorithm, which results in similar, but still different semantic profiles when starting from different random initializations. Using the same rank (*k *= 8 for the SGD8 set), we ran the NMF procedure 10 times, creating a set of 10 distinct factorizations. This gives rise to a new matrix of 575 genes and 80 semantic features (corresponding to 8 factors of 10 independent NMF experiments). We note that this vector representation is about *30 times smaller *than the initial term-based vector space representation (from an initial basis of 2,365 terms to 80 semantic features). It is important to note that the semantic features obtained by independent factorizations might be redundant, which implies the retention of the most stable sub-topics found within the literature corpus.

The combined use of semantic profiles obtained by 10 NMF experiments improves the reproducibility of results. This is shown by the increase of the correlation coefficient of the pair-wise gene distances built in two independent analyses. Using the combination of profiles obtained with 10 factorizations the correlation coefficient is 0.87, in contrast to correlations in the range of 0.40 for the distances obtained from single NMF factorizations.

**Figure 3:**
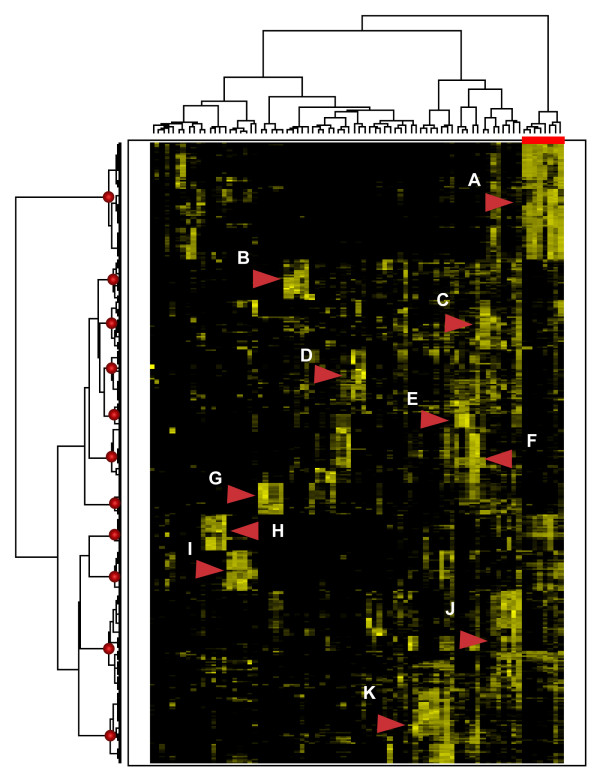
**SGD8 dataset gene clustering**. Two-way hierarchical clustering of both gene-documents and semantic features of the SGD8 set allows determination of gene clusters and corresponding significant factors.

Clustering of both genes and features in the semantic profiles allows us to analyze the set of semantic features that are relevant for each gene subset, as well as to evaluate their usefulness for the functional interpretation of the clusters. Cluster analysis can be done using any available clustering method. In this work we chose to use agglomerative hierarchical clustering (using Ward's algorithm and half square Euclidean distance) due to its simplicity and interpretability. Semantic profiles are first normalized by z-score to make independent NMF factorizations comparable. Results of hierarchical two-way clustering of SGD8 semantic profiles are provided in Figure 3, showing a threshold cutoff of 11 gene clusters (see also Additional file 2). The use of combined semantic profiles obtained from several independent NMF runs allowed for a more robust clustering, as independent factorizations provide insight into diverse biological themes.

Correspondence among the eleven gene clusters and the 80 semantic features for the SGD8 dataset is also indicated in Figure 3 (as shown for cluster B using the dotted lines in the figure). Characteristic semantic features for each cluster were constructed by averaging the individual member features. These average features reveal the most important terms for each coherent group. Subsequently, we asked the four experts to associate each of the average features (10 top terms) with the eight GO Slim categories, as well as to provide a free-text label (see table 4).

**Table 4:** SGD8 gene clusters. Clusters obtained from the SGD8 dataset.

***Cluster (num. genes)***	**Majority Slim annotation**	**Comments**
*A. DNA repair and replication (108 genes)*	'DNA metabolism' (103 genes)	The rest of the genes in the cluster (5) are also related to DNA repair and replication processes taking into account their functional annotations in SGD.
*B. Lipid metabolism (38 genes)*	'lipid metabolism' (32 genes)	Genes annotated with other Slim categories (6), also contain functional annotations in SGD revealing their implication in lipid metabolism.
*C. Response to stress (46 genes)*	'response to stress' (23 genes)	Among genes with other Slim categories there are genes involved in the ubiquitin-dependent protein catabolism (*GRR1, SKP1, CDC4, MET30, CDC34, CDC53, HRT1, UFD1, CDC48, RPN4, DOA1*); chaperones (SIS1, SSB1, YDJ1, SSA1) and RAS protein signal transduction (*RAS1, IRA2, CDC25*).
*D. Transport I (47 genes)*	'transport' (37 genes)	Most genes in the cluster (40) are annotated with membrane related localizations in GO cell component category: 'plasma membrane' (35 genes), 'periplasmic space' (4 genes) and 'membrane fraction' (1 gene).
*E. Transport II (25 genes)*	'transport' (20 genes)	13 genes correspond to hydrogen-transporting V-type ATPases (namely *STV1, VMA2, VPH1, VMA13, VMA8, VMA7, VMA5, TFP3, VMA6, VMA10, VMA4, TFP1, PPA1*). It also other 'transport' category proteins: four members of the P-type ATPase superfamily (*PMC1, PMR1, ENA1, PMA2*), and three ion channels (*CCH1, MID1 *and *FPS1*).
*F. Transport III (51 genes)*	'transport' (42 genes)	Non-transport genes are related to vacuole organization and inheritance (*FAB1, TRX1, TRX2*) or glycosylation (*MNN4, KRE2, WBP1*).
*G. Mitochondria (30 genes)*	'transport' (24 genes)	Contains mitochondria located genes. Transport genes: members of the mitochondrial protein translocase family (*TIM22, MRS11, TIM13, TIM8, TIM9, TIM17, MAS6, TOM40, TIM44, MGE1, TOM70, TOM20, TOM5, TOM22, TOM6, TOM7 *and *MRS5*); mitochondrial outer membrane porin (*POR1*), translocase of the mitochondrial inner membrane (*OXA1*). Non-transport genes are located in the 'mithocondrial matrix' (*SSQ1, HSP78, PIM1, PET54*), 'mitochondrial inner membrane' (*PET111*) and 'mitochondrion' (*MTF2*).
*H. Gene expression (chromatin) (33 genes)*	'DNA metabolism' (28 genes)	All genes contain chromatin related GO annotations in SGD. Contains 5 genes with other Slim categories related to chromatin.
*I. Cell cycle (37 genes)*	'cell cycle' (33 genes)	Contains also 'transport' and two "signal transduction" Slim genes. Transport genes are: *MAD1 *(annotated as mitotic spindle checkpoint) and *PDS1 *(essential for cell cycle arrest in mitosis in the presence of DNA damage or aberrant mitotic spindles).
*J. mRNA and protein biosynthesis (93 genes)*	'protein biosynthesis' (40 genes)	Other genes in the cluster include translation elongation and translation initiation factors as well as those involved in mRNA processing like mRNA catabolism, mRNA-nucleus export or the RNA polymerase II transcription machinery (e.g. regulators like *CDC36*, *CDC39*).
*K. Cell morphology response (67 genes)*	'cell wall organization and biogenesis' (25 genes)	Among them a significant number is related to cell shape and structure (cell wall and cytoskeleton), as well as events and processes related to morphological changes in the cellular envelope (cell budding, sporulation, conjugation with cellular fusion, endocytosis).

In the next two sections we evaluate the ability of our method to identify functionally coherent gene subsets, by detailed analysis of the eleven clusters obtained for the SGD8 set, as well as by comparing our results with the classification based on GO Slim categories.

### Detailed analysis of classes

We assess the performance of our method for the creation of functionally coherent gene subsets, as well as its capability to provide a justification of such categorization. Any expert-based categorization of genes is expected to yield somewhat different results, depending on the criteria used for the classification, as well as on the judgment and evidence used to assign a gene to a category. We note that our method is not driven by an *a priori *classification criterion, but relies on the automatic discovery and creation of similarity-based groups of genes constructed from a literature corpus analysis.

The analysis of the 575 genes in the SGD8 set resulted in eleven gene subsets (see table 5). Clusters A, B and C contain genes highly associated with '*DNA repair and replication*', '*lipid metabolism*' and '*response to stress*', respectively.

**Table 5:** Semantic features (Reelin dataset clusters). Top 10 terms of semantic features representing the four clusters obtained for the Reelin dataset. An average semantic feature has been calculated from the characteristic features in each cluster obtained by two-way hierarchical clustering.

**A. Cancer**	**B. Development**	**C. Alzheimer**	**D. Reelin**
p53	notch	app	tgf-beta
egfr	sonic	abeta	reelin
c-myc	notch1	amyloid	tau
breast	presenilin	gamma-secretas	fyn
tumor	tgf-beta	alzheim	egfr
cancer	limb	presenilin	phosphoryl
neu	bud	apo	apo
tgf-beta	ventral	beta-amyloid	src
p21	mesenchym	amyloid-beta	neuron
vegf	patch	plaqu	apolipoprotein

Clusters D, E and F constitute three separate groups of genes associated with '*transport*'. Although the terms comprising each average semantic feature do not provide a clear topical distinction between these three clusters, their existence reveals different aspects of transport within each of them. These aspects have been assessed by the analysis of functional annotations and descriptions of the genes in each cluster. Cluster D (*Transport I*) appears to group together genes associated with plasma membrane-related transport mechanisms, as most genes in the cluster are assigned GO annotations that indicate membrane related localizations by the SGD. Cluster E (*Transport II*) contains, among others, a significant number of V-type and P-type ATPases. Finally, genes in cluster F (*Transport III*) are related to autophagy (*ATG4*, *ATG5*, *ATG7*, *ATG8*, *ATG9 *and *ATG12*), ER to Golgi transport (vesicle-mediated transport), protein-vacuolar targeting and protein translocation (*SEC61*, *SEC63 *complexes and signal recognition particle (SRP)).

Cluster G (*mitochondria*) contains genes with mitochondrial localizations (including a significant number of mitochondrial transport genes). Cluster H (*gene expression- chromatin*) groups together genes with chromatin related GO annotations. Cluster I contains cell-cycle genes. Cluster J (*mRNA *and *protein biosynthesis*), includes translation elongation and translation initiation factors, as well as genes involved in mRNA processing.

**Figure 4:**
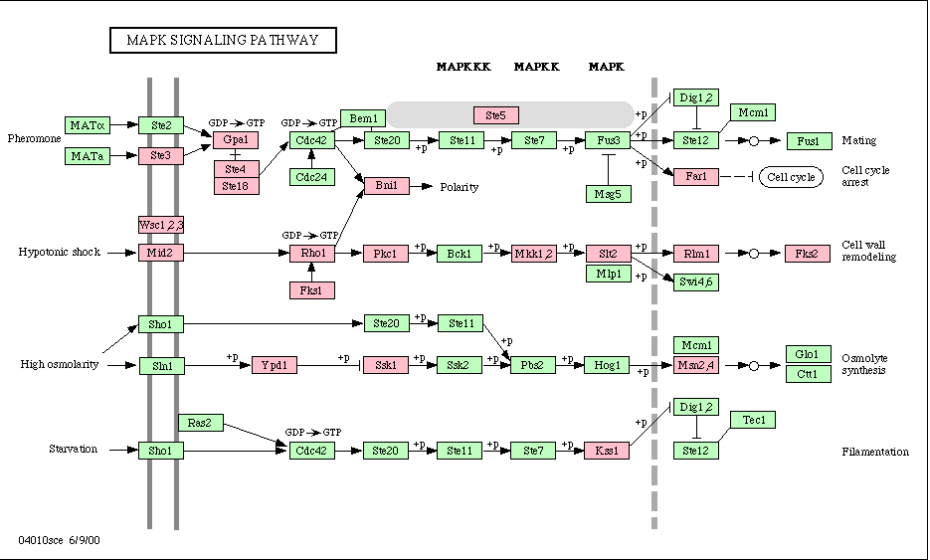
**MAPK signaling pathway mapping**. Subset of genes in cluster K (colored in pink) mapped onto the MAPK signaling pathway diagram for *S. cerevisiae *(04010sce pathway), as provided in the KEGG PATHWAY database [37].

Finally, cluster K (*cell morphology response*) contains a significant number of genes related to cell shape and structure, as well as those related to events and processes typically associated with morphological changes in the cellular envelope (cell budding, sporulation, conjugation with cellular fusion, endocytosis). A closer look at these genes allows us to observe the existence of a significant number related to the MAP kinase signaling pathway. The MAPK cascade is related to various cellular processes: the pheromone response pathway, filamentous invasive growth, hyperosmotic response and cell wall remodeling. We have found genes in this cluster related to all of these networks: *STE5*, *STE4*, *STE8*, *STE3*, *GPA1*, *FAR1 *(pheromone pathway); *WSC2, MID2, RHO1, FKS1, PKC1, MKK1, MKK2, SLT2, RLM1, FKS2 *(cell wall remodeling); *YPD1, SSK1, MSN2, MSN4, PTC2, STE50 *(hyperosmotic response); *KSS1, PFY1, VRP1 *(filamentous growth). Figure 4 shows those genes mapped to the *S. cerevisiae *MAPK signaling pathway as provided by the KEGG PATHWAY database [35-37].

### What happened to the original GO Slim categories?

As demonstrated by the above analysis, the gene relationships built by our proposed methodology correspond well to biologically relevant information. Nevertheless, the SGD8 set was originally constructed from only eight broad process categories annotated by experts, while our method produced eleven clusters. At this point, we assess the relationships between the eight Slim categories and the eleven functional clusters obtained by our methodology.

The GO Slim category '*lipid metabolism*' maps clearly to cluster B (*lipid metabolism*), as 94.1% of genes annotated with the category 'lipid metabolism' are grouped together in this cluster, constituting 84% of the genes in the cluster. Our method has also discovered the relationship of six additional genes, annotated by other Slim categories, to lipid metabolism, based on their associated literature.

Two Slim categories ('*DNA metabolism*' and '*transport*') are subdivided by our method into more specific sub-categories. Nearly 90% of 'DNA metabolism' Slim genes are found in two clusters (A and H). Among these genes, those assigned to Cluster A (*DNA repair and replication*) account for 95.4% of the cluster while those in cluster H (*gene expression-chromatin*) account for 84.8% of the genes in the cluster. In both cases, the method has been able to provide a justification of these specializations through the semantic features that link the two clusters to more specific '*DNA repair and replication*' and '*gene expression-chromatin*' subtopics within the '*DNA metabolism*' broader category.

Similarly, most of the Slim '*transport*' genes (80.9%) are placed by our method in four clusters: cluster D (*transport I*) (78.7%), cluster E (*transport II*) (80%), cluster F (*transport III*) (82.3%) and cluster G (*mitochondria*) (80%). This division of the initial 'transport' category into more specific, coherent subgroups is specifically exemplified by cluster D, which contains transport proteins located in the plasma membrane and periplasmic space. Additionally, cluster G demonstrates an organelle-based criterion for categorization, as it groups genes encoding mitochondrial located proteins.

Four Slim categories ('*cell cycle*', '*response to stress*', '*protein biosynthesis*' and '*cell wall organization and biogenesis*') have been somewhat redefined. In the case of the '*cell cycle*' and '*response to stress*' categories, the genes that are kept clustered together account for less than half of their new clusters (42.8% in cluster I and 36.5% in cluster C respectively). However, while '*cell cycle*' genes are representative of cluster I (89%), 'response to stress' genes form just 50% of cluster C. This means that 'cell cycle' category has been reduced by specification (only retaining the most specific genes).

In contrast, 63.5% of the 'response to stress' genes have been reassigned to different clusters, although they are not representative of any of them. A possible explanation for this reassignment is that it may be difficult to keep a homogeneous and generic 'response to stress' group based on the biomedical literature corpus analyzed. Since the cellular response to stress triggers a multitude of cellular processes and biochemical mechanisms, any classification could potentially relate 'response to stress' genes to any of these processes. This is the case of the SGD8 dataset, where our method identified salient features related to protein degradation in a subset of the data which are the basis for cluster C. The rest of genes annotated with Slim 'response to stress' have been grouped in several clusters according to different criteria. E.g. *TEL1, SML1, LCD1 *involved in the response to DNA damage have been assigned to cluster A (*DNA repair and replication*); DPL1 implicated in sphingolipid metabolism to cluster B (*lipid metabolism*); protein kinase CK2 complex proteins (*CKA1, CKA2, CKB1, CKB2*) related to a corresponding modified ribosome P2 protein (Rpp2Ap) are placed in cluster J (*mRNA and protein biosynthesis*).

In the case of '*protein biosynthesis*' and '*cell wall organization and biogenesis*' most genes (81.6% and 78%) remain together in the same group although they represent less than half of the genes in their respective clusters (43% in cluster J and 47,7% in cluster K). The reason for these changes is the functional characteristics that are assigned by our method to the new clusters. In our results, cluster J includes not only genes related to protein biosynthesis, but also mRNA biosynthesis and degradation. Along the same lines, cluster K includes cell wall genes as well as other genes related to processes requiring changes in cell morphology. Note that these characteristics of the clusters are indicated by the terms associated with their semantic features as shown in Table 3.

**Table 3:** Semantic features (SGD8 dataset clusters). Top 10 terms of semantic features representing the eleven clusters obtained for the SGD8 dataset. An average semantic feature has been calculated from the characteristic features in each cluster obtained by two-way hierarchical clustering.

**A. DNA repair and replication**	repair, dna, replic, telomere, checkpoint, pcna, damag, dsb, recombin, mismatch
**B. Lipid metabolism**	sterol, fatti, lipid, ergosterol, synthetas, synthas, biosynthesi, actin, heat, sphingolipid
**C. Response to stress**	mitochondri, hsp70, ubiquitin, dna, oxid, chaperon, shock, rad52, heat, camp
**D. Transport I**	transport, membran, vesicle, golgi, outer, receptor, copii, export, snare, vacuolar
**E. Transport II**	vacuolar, v-atpas, membran, vacuol, vesicl, transport, golgi, glucose, transloc, cytosol
**F. Transport III**	transport, uptake, vesicle, vacuolar, vacuole, membran, permeas, nitrogen, ubiquitin, glucos
**G. Mitochondria**	mitochondria, mitochondri, mitochondrion, inner, ino1, matur, translat, transles, membrane-associ, membran
**H. Gene expression (chromatin)**	nucleosom, histon, swi, snf, chromatin, remodel, arrai, transcript, silenc, acetyl
**I. Cell cycle**	spindl, cyclin, kinetochor, checkpoint, anaphase, mitosi, mitot, sister, chromosom, replic
**J. mRNA and protein biosynthesis**	translat, mrna, ribosom, rna, poli, swi, snf, elong, transcript, atpas
**K. Cell morphology response**	actin, kinas, wall, phosphoryl, pheromone, mate, phosphates, cytoskeleton, glucose, polar

Finally, genes that were Slim annotated with '*signal transduction*' have been assigned to several clusters, causing the dilution of this process category among other biological topics in the set. As in the case of '*response to stress*', signal transduction is a broad category that covers several physiological processes and biological pathways. Our analysis has not found semantic features common to all 'signal transduction' genes in the SGD8 set that allow them to cluster together. However, the semantic features discovered can relate some signaling genes to still relevant processes. For example, MAPK cascade genes in cluster K (*cell morphology response*), or *DPP1 *and *LCB3 *in cluster B (*lipid metabolism*).

### Robustness of the method

In the experiments reported above we used curated, relevant documents. To demonstrate that our method can perform well even when the literature corpus contains a certain degree of irrelevant documents, we performed an additional experiment. We added 10% of noisy documents to the list of references for each gene. These noisy documents where picked at random from among the MEDLINE documents published in English between the years 2000 and 2004, whose MESH annotation include the three terms: 'genes', 'proteins' and 'Saccharomyces cerevisiae'. This selection of documents ensures that while the documents may not all be relevant to the gene set, they still contain similar terms similar to those in the original corpus.

Distance values among genes (one minus cosine of the angle between gene-documents) calculated from the noisy corpus were compared to those obtained using the original corpus with a correlation coefficient of 0.83, demonstrating that the method is able to find similarities among genes even in the presence of 10% irrelevant references per gene. The differences are obviously associated with the unrelated information provided by the irrelevant references; however, the high correlation coefficient demonstrates that our approach is able to extract the main structure of the data, even in the presence of noise.

### Reelin dataset

In addition to the 575 genes in the SGD8 dataset, we have tested our method on a data set that was recently used by Homayouni *et al. *(2005). They used latent semantic indexing of article abstracts (based on singular value decomposition, SVD) to analyze a set of 50 genes. The 50-gene set is based on the manual selection of genes related to cancer biology, Alzheimer's disease and development, and includes 5 genes that are involved in the Reelin pathway. As the exact set of documents used by Homayouni *et al*. is not readily available, we have analyzed the 50 gene set provided by them, using the literature relevant to the human and mouse genes as obtained from Entrez Gene [32], following the procedure described by Homayouni et al. (2005).

**Figure 5:**
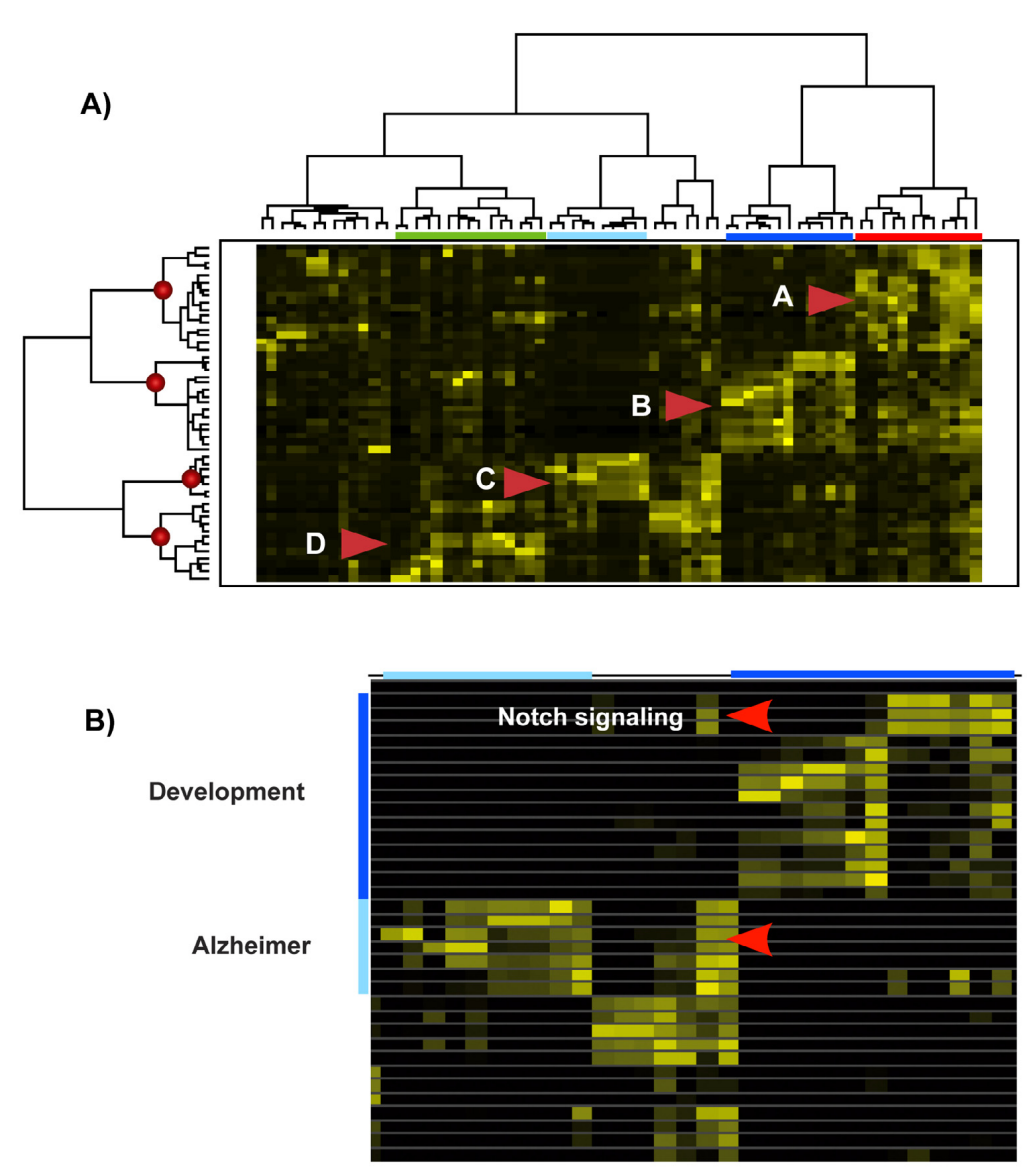
**Reelin dataset gene clustering**. A) Two-way hierarchical clustering of the Reelin set corresponding to 10 NMF factorizations with *k *= 7. Four cluster selection. B) Detailed view, where the semantic feature common to Notch signaling genes and *Alzheimer *cluster is highlighted.

Our method was applied as described for the SGD8 set. From a literature corpus of 4,378 distinct articles, a matrix of 50 genes and 1,865 terms was obtained (terms were included if found in at least 10 and no more than 40 genes). Factorization rank *k *= 7 was selected, as before, using the cophenetic coefficient (see Additional file 1). The result from clustering the gene semantic profiles is shown in Figure 5 (using a 4 cluster threshold in the dendrogram). Complete information about the results can be found in Additional file 2.

Four clusters were established labeled as *cancer, development, Alzheimer *and *Reelin *respectively. Corresponding semantic features are provided in table 5. Cluster A (*cancer*) contains all the genes annotated as such by Homayouni *et al*., together with *TGFB1 *and *WNT2 *(*development *and *cancer*). Cluster B (*development*) contains all the development and cancer genes with the exception of *TGFB1 *(which is in cluster A), together with *ATOH1 *(annotated as '*development*'). As expected, most genes in this cluster also have high values for semantic features associated with cluster A (*cancer*), since all the genes except *ATOH1 *were also annotated with the '*Cancer*' category by the original authors. Among the genes in cluster B (*development*), it is interesting to note a subgroup related to Notch signaling (*NOTCH1, JAG1 *and *DLL1*) with a clear differentiated semantic profile. Cluster C (*Alzheimer*) contains some of the Alzheimer genes (namely *APLP1, APLP2, APBA1, APBB2, APP, PSEN1 *and *PSEN2*). Finally, Cluster D (Reelin) contains the five Reelin pathway genes in the set, as well as development & Alzheimer genes (*CDK5, CDK5R, CDK5R2*), along with a subset of Alzheimer genes (namely *MAPT, A2M, APOE *and *LRP1*).

The results of our analysis clearly show that, although the Reelin pathway genes are clustered together with some known Alzheimer's disease genes, they are not the only ones that share semantic features with Alzheimer's-disease-associated genes. Careful examination of the semantic features shows putative connections between the Alzheimer-implicated genes and other development genes. This is the case of the Notch signaling genes in the set (namely *NOTCH1, JAG1 *and *DLL1*), grouped in cluster B, that also have strong signatures of semantic features which are high in some of the genes in cluster C (*Alzheimer*). Hints of these connections are provided by shared features of Notch signaling genes with cluster C, as shown in Figure 5b (*apo, notch, tau, app, abeta, presenilin, apolipoprotein, gamma-secretas, alzheim, amyloid*).

It is important to note that in contrast to SVD, the nonnegative constraints imposed in NMF, make the representation of genes as an additive combination of semantic features directly interpretable, as combinations of sets of terms. Therefore, in addition to the categorization of genes, our method also provides valuable clues about the semantics of the relations underlying the resulting clusters. These clues are given by the terms characterization associated with each cluster. For a further comparison of Reelin dataset clustering using profiles obtained by SVD and NMF see Additional file 4.

## Discussion

The ultimate goal of text mining is to discover and derive new information from textual data, finding patterns across datasets, and separating signal from noise [3]. In this work we propose a text mining method that is able to find semantic features from the literature corpus relevant to a set of biological entities (specifically, genes or proteins). These semantic features form a basis by which genes and proteins are represented in the form of semantic profiles. Both the features and the profiles are simultaneously inferred during the learning process. Therefore, the profile created for a particular gene will be suited to the context of the particular gene set analyzed. The method relies on the use of non-negative matrix factorization (NMF), which is a machine-learning algorithm that has been previously applied to document clustering [27-29]. This new semantic space representation allows relating genes or proteins using profile similarity measures, while directly providing means for interpreting large sets of experimental data. In addition, the reduced dimensionality of the semantic space makes this representation amenable to integration with experimental measurements (e.g. gene expression data).

Semantic profiles obtained by our method provide several advantages over literature profiles obtained using previous approaches [12,15,19], as they combine the best properties found in several models:

• *Low-dimensionality*, similar to SVD, but contrasted with the classical vector space model, NMF aims to represent the high dimensional text data in a much lower dimensional space. The basic idea is to approximate the original data matrix by the product of two, or more, matrices of lower rank. There are known advantages to reduced dimensionality, as noted in the context of the well-studied vector space model (terms-documents frequency matrix), in which representations are typically both very large and quite sparse. High-dimensional vectors make for highly inefficient data analysis, and the quality of the results is easily affected by noisy and sparse data.

• *Latent semantics*. NMF, again like SVD, is an approach for performing latent semantic analysis (LSA) [27]. LSA techniques have been widely applied within information retrieval [38,39]. As in other LSA techniques, the relation established by NMF between terms is not the relative frequency with which they tend to co-occur, but the extent to which they have the same effect in the construction of total passage meanings [40]. In addition, NMF can alleviate polysemy, disambiguating meanings in the corpus of documents [22].

• *Non-orthogonality*. In contrast to the widely-used SVD, NMF does not enforce the production of orthogonal features. The requirement of orthogonal features imposed by SVD leads to features which do not naturally correspond to each of the original term sets [27]. Usually topics described in a literature corpus are not completely independent of each other, and there can be some overlap among them. In such a case, the axes of the semantic space that capture each of the topics are not necessarily orthogonal. The orthogonal features produced by SVD, whose linear combination can reproduce the documents, thus lose the intuitive meaning of sets of terms (see the point about interpretability below). In this sense, NMF is particularly well-suited for capturing relationships that underlie highly connected biological processes.

• *Interpretability*. The most significant drawback of LSA via singular value decomposition (SVD) is the lack of the interpretability of the low-dimensional features. The SVD-resulting features are no longer term associations but numbers in some low-dimensional space. Contrary to SVD, NMF imposes non-negativity constraints on both the basis and encoding vectors. These constraints lead to a part-based representation of data as they allow only additive, not subtractive combinations [22]. As a result, both the features and their contribution to gene profiles can be readily interpreted as a combination of their most significant terms. Additionally, the features obtained by NMF tend to be sparse in the sense that the most important features are reinforced while less important ones are diminished, giving rise to local features that correspond to "parts" in the data. These local features can be directly interpreted since they contain the most significant terms appearing together in the corpus and thus semantically related.

Our approach requires a collection of literature references relevant to each gene in the set; it does not require precompiled thesaurus, vocabularies or experimental-based hypothesis. In this respect it is similar to earlier work by [12,15,19]. The availability of gene-relevant documents is currently a limitation of these text mining methods. Nevertheless, there are two strong areas of work that support their realistic use in the present and near future. On one hand, ongoing efforts in database annotation are providing an increasing number of bibliographic references. On the other, the development of automatic methods to perform gene document retrieval is a very active area of research, from which we can expect improvements in precision ratios. These methodologies rely on the premise that similarity among genes can be established by the similarities found in texts describing their biological roles (i.e. their associated documents). As these methods use bag-of-words models, the nonlinear intra-sentence syntactic and grammatical effects on meaning, such as predication, attachment, negation, and propositional implication are lost [40]. However, the use of several abstracts to represent each gene via the construction of a composite gene-document reduces this effect. Multiple abstracts representing each gene help expose the strong relationships among concepts which are expressed in different ways throughout the abstracts, while arbitrary connections are weakened by averaging.

Literature relevant to a given gene might discuss several functional roles of the gene in the cell. The method presented in this work directly allows for genes to be associated with multiple topics. As genes are represented as additive linear combinations of a reduced number of semantic features, researchers can also explore similarities in literature profiles interactively (an example of a gene represented as term-vector, cluster-vector and semantic profile is provided in Additional file 3).

In this study, we have tested the performance of our method on two different gene collections, for which a minimum number of relevant bibliographic references were available. In both cases we were able to provide gene literature profiles as combinations of the common semantic features discovered from the literature corpus. We have also verified the value of semantic features as cues for biological interpretation of gene profiles. Finally, we have analyzed the semantic profiles to establish coherent groups of genes. The results obtained by gene profile clustering are consistent with commonalities in expert-based annotations, and additionally have revealed relationships that could not have been recognized by the analysis of the functional annotations alone. In addition, our results shown that the method is robust in the presence of 10% additional irrelevant documents per gene. Nevertheless, further work is needed to test the method in an experimental setup where curated references are not available, and current state-of-the-art document retrieval applications are therefore used to create the literature corpus.

Our test sets are of the same order of magnitude previously used in quite a few studies using related text mining approaches in biomedical informatics, and are of valid size for practical applications. We note though that showing the applicability to larger sets of genes is an important extension. In this sense, we note that the computational complexity of the classical NMF algorithm [22] is O(*kpn*) per iteration, where *k *is the number of features, *p *is the number of terms and *n *is the number of documents [28]. In addition, several implementations to support speedup have been proposed [41], as well as variations to exploit different characteristics of the new representation space (e.g. the sparsity level [42]). Moreover, while further optimization is beyond the scope of this paper, NMF uses a well-defined functional whose optimization can be improved in several ways. We are currently experimenting with sets of genes that are an order of magnitude larger (thousands of genes), and working on scaling up the algorithms themselves to support fast, large-scale computations.

## Conclusion

We have presented a method that is able to discover semantic features from the analysis of literature relevant to sets of genes. The representation of genes as additive linear combinations of basis semantic features allows for the exploration of functional associations as well as clustering. We anticipate the potential use of our method for the validation and interpretation of high-throughput experimental data, as well as for the analysis of any genome-wide information.

## Methods

### Constructing gene-documents

A document is constructed for each gene by concatenating the titles and the abstracts of all its relevant bibliographic references. Specifically, each gene-document is represented by a weighted term vector, as in the classical vector space model used in information retrieval (IR) systems [13,14]. Under this model a document (*i*) is represented as a vector of term-weights of the form **D**_*i *_∈ ℝ^*p*^, where *p *represents the total number of terms in the vocabulary of the text corpus, and each element *D_ij _*in the vector is a weight representing the relative importance of the *j*^th ^term in the document *i*. The definition of a term is not inherent in the model, but terms are often chosen to be single words.

Typically, the weight is directly proportional to the term frequency within the document, and invertly proportional to other factors which may reduce its importance, (e.g. the total length of the document, the abundance of the term throughout the corpus, etc.). Various weighting methods have been developed in the information retrieval arena [43]. The scheme most often used is known broadly as TF*IDF, where TF stands for *term frequency*, and IDF stands for *inverse document frequency *[44]. This weighting scheme discounts the importance of terms that appear in many documents and are thus not strongly indicative of a document's content. Formally, the IDF for the *j*^th ^term is calculated as:

idfj=log⁡(Ttj)     Eq. 1
 MathType@MTEF@5@5@+=feaafiart1ev1aqatCvAUfKttLearuWrP9MDH5MBPbIqV92AaeXatLxBI9gBaebbnrfifHhDYfgasaacH8akY=wiFfYdH8Gipec8Eeeu0xXdbba9frFj0=OqFfea0dXdd9vqai=hGuQ8kuc9pgc9s8qqaq=dirpe0xb9q8qiLsFr0=vr0=vr0dc8meaabaqaciaacaGaaeqabaqabeGadaaakeaacqWGPbqAcqWGKbazcqWGMbGzdaWgaaWcbaGaemOAaOgabeaakiabg2da9iGbcYgaSjabc+gaVjabcEgaNnaabmaabaWaaSaaaeaacqWGubavaeaacqWG0baDdaWgaaWcbaGaemOAaOgabeaaaaaakiaawIcacaGLPaaacaWLjaGaaCzcaiabbweafjabbghaXjabb6caUiabbccaGiabbgdaXaaa@4384@

where *T *is the total number of documents in the set (total number of gene-documents in this case), and *t_j _*is the number of gene-documents that contain the term *j*.

Thus the weight assigned to term *j *in document *i *under the TF*IDF scheme is:

*D_ij _= tf_ij_·idf_j _*    Eq. 2

In text analysis, common words (also known as *stop words*) are typically eliminated from texts prior to the calculation of term frequencies. Additionally, word morphological variants are reduced to their root form using the Porter stemming algorithm [45].

To summarize, a set of *n *gene-documents, over a vocabulary of *p *terms, is represented as a set of *n *vectors in *p*-dimensional space, where *p *is typically very high. Therefore, every term in the corpus vocabulary becomes an independent dimension in a very high dimensional space. Since each document contains only a limited set of terms (compared to the whole corpus term collection), most of the vectors are very sparse.

### Dimensionality reduction: extracting semantic features

Once the gene-document collection is represented in a vector space model, as an *p *× *n *sparse matrix, the next step is to find relevant common sub-sets of terms that correspond to latent concepts in the literature corpus. This is accomplished through the application of non-negative matrix factorization [22].

Formally, the non-negative matrix factorization (NMF) is described as follows:

**V ≈ WH **    Eq. 3

where **V **∈ ℝ^*p × n *^is a positive data matrix with *p *variables and *n *vectors, **W **∈ ℝ^*p × k *^are the reduced *k *basis vectors or factors, and **H **∈ ℝ^*k × n *^contains the coefficients of the linear combinations of the basis vectors needed to reconstruct the original data (also known as encoding vectors). Additionally we have the following conditions: *k *≤ *p*, all matrices **V, W, H **are non-negative, and the columns of **W **(the basis vectors) are normalized (sum to 1).

The main difference between NMF and other classical factorization models (e.g. SVD) lies in the nonnegativity constraints imposed on both the basis (**W**) and encoding vectors (**H**). In this way, only additive combinations are possible:

(V)iμ≈(WH)iμ=∑a=1kWiaHaμ     Eq. 4
 MathType@MTEF@5@5@+=feaafiart1ev1aaatCvAUfKttLearuWrP9MDH5MBPbIqV92AaeXatLxBI9gBaebbnrfifHhDYfgasaacH8akY=wiFfYdH8Gipec8Eeeu0xXdbba9frFj0=OqFfea0dXdd9vqai=hGuQ8kuc9pgc9s8qqaq=dirpe0xb9q8qiLsFr0=vr0=vr0dc8meaabaqaciaacaGaaeqabaqabeGadaaakeaacqGGOaakcqWHwbGvcqGGPaqkdaWgaaWcbaGaemyAaKgcciGae8hVd0gabeaakiabgIKi7kabcIcaOiabhEfaxjabhIeaijabcMcaPmaaBaaaleaacqWGPbqAcqWF8oqBaeqaaOGaeyypa0ZaaabCaeaacqWGxbWvdaWgaaWcbaGaemyAaKMaemyyaegabeaakiabdIeainaaBaaaleaacqWGHbqycqWF8oqBaeqaaaqaaiabdggaHjabg2da9iabigdaXaqaaiabdUgaRbqdcqGHris5aOGaaCzcaiaaxMaacqqGfbqrcqqGXbqCcqqGUaGlcqqGGaaicqqG0aanaaa@5274@

The objective function, based on the Poisson distribution, can be defined using the following divergence function, which the factorization process needs to minimize:

D(V,WH)=∑i=1p∑j=1n(Vijln⁡Vij(WH)ij−Vij+(WH)ij)     Eq. 5
 MathType@MTEF@5@5@+=feaafiart1ev1aaatCvAUfKttLearuWrP9MDH5MBPbIqV92AaeXatLxBI9gBaebbnrfifHhDYfgasaacH8akY=wiFfYdH8Gipec8Eeeu0xXdbba9frFj0=OqFfea0dXdd9vqai=hGuQ8kuc9pgc9s8qqaq=dirpe0xb9q8qiLsFr0=vr0=vr0dc8meaabaqaciaacaGaaeqabaqabeGadaaakeaacqWGebardaqadaqaaiabhAfawjabcYcaSiabhEfaxjabhIeaibGaayjkaiaawMcaaiabg2da9maaqahabaWaaabCaeaadaqadaqaaiabdAfawnaaBaaaleaacqWGPbqAcqWGQbGAaeqaaOGagiiBaWMaeiOBa42aaSaaaeaacqWGwbGvdaWgaaWcbaGaemyAaKMaemOAaOgabeaaaOqaamaabmaabaGaeC4vaCLaeCisaGeacaGLOaGaayzkaaWaaSbaaSqaaiabdMgaPjabdQgaQbqabaaaaOGaeyOeI0IaemOvay1aaSbaaSqaaiabdMgaPjabdQgaQbqabaGccqGHRaWkdaqadaqaaiabhEfaxjabhIeaibGaayjkaiaawMcaamaaBaaaleaacqWGPbqAcqWGQbGAaeqaaaGccaGLOaGaayzkaaaaleaacqWGQbGAcqGH9aqpcqaIXaqmaeaacqWGUbGBa0GaeyyeIuoaaSqaaiabdMgaPjabg2da9iabigdaXaqaaiabdchaWbqdcqGHris5aOGaaCzcaiaaxMaacqqGfbqrcqqGXbqCcqqGUaGlcqqGGaaicqqG1aqnaaa@694B@

To solve the optimization problem posed by equation 5, the following iterative algorithm is used:

1. Initialize **W **and **H **with positive random numbers.

2. For each basis vector **W**_*a *_∈ ℝ^*p *× 1^, update the corresponding encoding vector **H**_*a *_∈ ℝ^1 × *n*^; followed by updating and normalizing the basis vector **W**_*a*_. Repeat this process until convergence.

The above iterative process converges to a local minimum of the objective function given in equation 5. The detailed algorithm follows:

Repeat until convergence:

For a = 1...*k *do begin

For b = 1...*n *do

Hab←Hab∑i=1p(WiaVib)/∑q=1kWiqHqb∑i=1pWia     Eq. 6
 MathType@MTEF@5@5@+=feaafiart1ev1aaatCvAUfKttLearuWrP9MDH5MBPbIqV92AaeXatLxBI9gBaebbnrfifHhDYfgasaacH8akY=wiFfYdH8Gipec8Eeeu0xXdbba9frFj0=OqFfea0dXdd9vqai=hGuQ8kuc9pgc9s8qqaq=dirpe0xb9q8qiLsFr0=vr0=vr0dc8meaabaqaciaacaGaaeqabaqabeGadaaakeaacqWGibasdaWgaaWcbaGaemyyaeMaemOyaigabeaakiabgcziSkabdIeainaaBaaaleaacqWGHbqycqWGIbGyaeqaaOWaaSaaaeaadaaeWbqaamaalyaabaWaaeWaaeaacqWGxbWvdaWgaaWcbaGaemyAaKMaemyyaegabeaakiabdAfawnaaBaaaleaacqWGPbqAcqWGIbGyaeqaaaGccaGLOaGaayzkaaaabaWaaabCaeaacqWGxbWvdaWgaaWcbaGaemyAaKMaemyCaehabeaakiabdIeainaaBaaaleaacqWGXbqCcqWGIbGyaeqaaaqaaiabdghaXjabg2da9iabigdaXaqaaiabdUgaRbqdcqGHris5aaaaaSqaaiabdMgaPjabg2da9iabigdaXaqaaiabdchaWbqdcqGHris5aaGcbaWaaabCaeaacqWGxbWvdaWgaaWcbaGaemyAaKMaemyyaegabeaaaeaacqWGPbqAcqGH9aqpcqaIXaqmaeaacqWGWbaCa0GaeyyeIuoaaaGccaWLjaGaaCzcaiabbweafjabbghaXjabb6caUiabbccaGiabbAda2aaa@67C4@

For c = 1...*p *do begin

Wca←Wca∑j=1n(HajVcj)/∑q=1kWcqHqj∑j=1nHaj     Eq. 7
 MathType@MTEF@5@5@+=feaafiart1ev1aaatCvAUfKttLearuWrP9MDH5MBPbIqV92AaeXatLxBI9gBaebbnrfifHhDYfgasaacH8akY=wiFfYdH8Gipec8Eeeu0xXdbba9frFj0=OqFfea0dXdd9vqai=hGuQ8kuc9pgc9s8qqaq=dirpe0xb9q8qiLsFr0=vr0=vr0dc8meaabaqaciaacaGaaeqabaqabeGadaaakeaacqWGxbWvdaWgaaWcbaGaem4yamMaemyyaegabeaakiabgcziSkabdEfaxnaaBaaaleaacqWGJbWycqWGHbqyaeqaaOWaaSaaaeaadaaeWbqaamaalyaabaWaaeWaaeaacqWGibasdaWgaaWcbaGaemyyaeMaemOAaOgabeaakiabdAfawnaaBaaaleaacqWGJbWycqWGQbGAaeqaaaGccaGLOaGaayzkaaaabaWaaabCaeaacqWGxbWvdaWgaaWcbaGaem4yamMaemyCaehabeaakiabdIeainaaBaaaleaacqWGXbqCcqWGQbGAaeqaaaqaaiabdghaXjabg2da9iabigdaXaqaaiabdUgaRbqdcqGHris5aaaaaSqaaiabdQgaQjabg2da9iabigdaXaqaaiabd6gaUbqdcqGHris5aaGcbaWaaabCaeaacqWGibasdaWgaaWcbaGaemyyaeMaemOAaOgabeaaaeaacqWGQbGAcqGH9aqpcqaIXaqmaeaacqWGUbGBa0GaeyyeIuoaaaGccaWLjaGaaCzcaiabbweafjabbghaXjabb6caUiabbccaGiabbEda3aaa@67D2@

Wca←Wca∑j=1nWja     Eq. 8
 MathType@MTEF@5@5@+=feaafiart1ev1aaatCvAUfKttLearuWrP9MDH5MBPbIqV92AaeXatLxBI9gBaebbnrfifHhDYfgasaacH8akY=wiFfYdH8Gipec8Eeeu0xXdbba9frFj0=OqFfea0dXdd9vqai=hGuQ8kuc9pgc9s8qqaq=dirpe0xb9q8qiLsFr0=vr0=vr0dc8meaabaqaciaacaGaaeqabaqabeGadaaakeaacqWGxbWvdaWgaaWcbaGaem4yamMaemyyaegabeaakiabgcziSoaalaaabaGaem4vaC1aaSbaaSqaaiabdogaJjabdggaHbqabaaakeaadaaeWbqaaiabdEfaxnaaBaaaleaacqWGQbGAcqWGHbqyaeqaaaqaaiabdQgaQjabg2da9iabigdaXaqaaiabd6gaUbqdcqGHris5aaaakiaaxMaacaWLjaGaeeyrauKaeeyCaeNaeeOla4IaeeiiaaIaeeioaGdaaa@4816@

End

End

For the application described here, a corpus of documents is summarized by a matrix **V**, which is obtained by transposing the matrix **D **defined in equation 2. Because of the sparsely distributed representation of NMF, each factor (column) in the matrix **W **is represented by a small subset of the terms, which constitutes a semantic feature in which NMF groups together topically related terms. Due to the positive nature of the model, these factors can be directly interpreted based on the most important terms they contain (highest values). Additionally, the analysis of the encoding vectors **H **(called the *semantic profiles*) provides information about the combination of topics or semantic features that describe each gene or protein.

An important consideration in the application of NMF to extract semantic features is the selection of the lower dimension, namely the factorization rank (*k*), to better represent the original data. Intuitively, the more factors we use, the more detailed information we get. However, since one of the main motivations for this application is the automatic summarization of the latent information in the scientific literature, it is important to obtain a reduced set of factors that are sufficient to represent the semantics, without obscuring this information with too many details. Finding an appropriate value of *k *depends on the application and on the nature of the dataset itself. This value is generally chosen such that (*n *+ *p*) *k *<*np *and thus the product **WH **can be regarded as a compressed form of the data in **V. **[22].

The selection of the factorization rank is a complex problem and different criteria can be used depending on the application. In this work, we followed the approach proposed by Brunet *et al*. (2004), and used the cophenetic correlation coefficient to choose a factorization rank that would best retain the stability of the results with respect to the different random initial conditions for **W **and **H**.

### Gene-document clustering

Once the dimensionality-reduction step is executed, gene-documents are represented as linear additive combinations of semantic features, which are called *semantic profiles.*

As described above, the NMF algorithm may not necessarily converge to the same solution on each run. The specific solution depends on the random initial conditions. We note though that having multiple solutions does not imply that any of them must be erroneous. This situation only indicates that the solution is not unique and can actually be exploited to our own advantage. In this application, a set of different solutions obtained from different random initial conditions typically produces semantic profiles that have many terms in common while slightly differ in others. These similar solutions represent semantic variations of gene-documents that are worth taking into account, as they may represent several functional aspects of the same gene. Therefore, to provide both a more comprehensive representation of the genes, and a more robust clustering, we constructed semantic profiles of gene-documents by combining the results from 10 independent runs of the NMF algorithm, using the same number of factors at each run. Thus, when using *k *factors, each gene is ultimately represented as a 10*k *dimensional vector over the semantic features. Cluster analysis is then performed on the total number of factors in order to discover gene relationship based on the semantic features representing them.

Clustering was carried out by means of agglomerative hierarchical clustering, using Ward's algorithm and half square Euclidean distance as a similarity measurement [46]. Two-way clustering of both genes and semantic features was performed in order to identify characteristic features for each gene cluster.

## Authors' contributions

MC and PCS carried out the computational studies and analysis, assessing and providing improvements to the method. HS and JMC revised both the methodology and manuscript critically for important intellectual content. APM designed and programmed the algorithms, as well as supervised and assessed the overall work. All authors participated in writing, approving and revising the final manuscript.

## Supplementary Material

Additional File 1 **Cophenetic correlation coefficient**. This file contains three graphs showing the cophenetic correlation coefficient for the SGD8, Reelin and random datasets. Click here for file

Additional File 2 **Clustering results**. This file contains two spreadsheets with the results of the clustering performed on the semantic profiles (also included after z-score normalization) obtained for the SGD8 and Reelin datasets. Click here for file

Additional File 4 **Reelin dataset analysis by SVD and NMF**. Descriptive comparison of the results obtained by clustering of gene profiles obtained by SVD and NMF algorithms. Includes hierarchical trees. Click here for file

Additional File 3 **Example representation of literature profiles**. This file contains the representation of a literature profile for gene *PET54 *as obtained in the vector space model, a clustered space and the semantic space. Click here for file
