# Supplementary figures and images for: Discovering semantic features in the literature: a foundation for building functional associations
Source: BMC Bioinformatics. 2006 Jan 26;7:41. doi: 10.1186/1471-2105-7-41 (PMC1386711; doi:10.1186/1471-2105-7-41)

# COPHENETIC CORRELATION COEFFICIENT

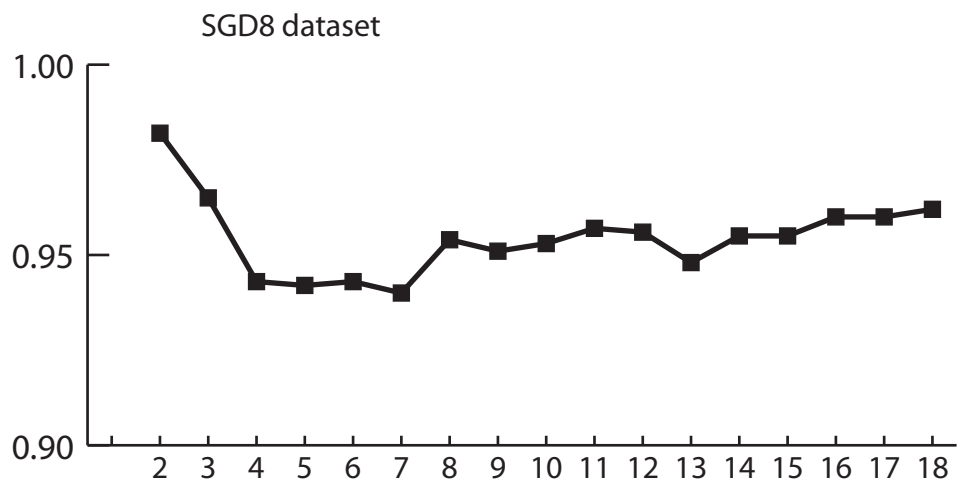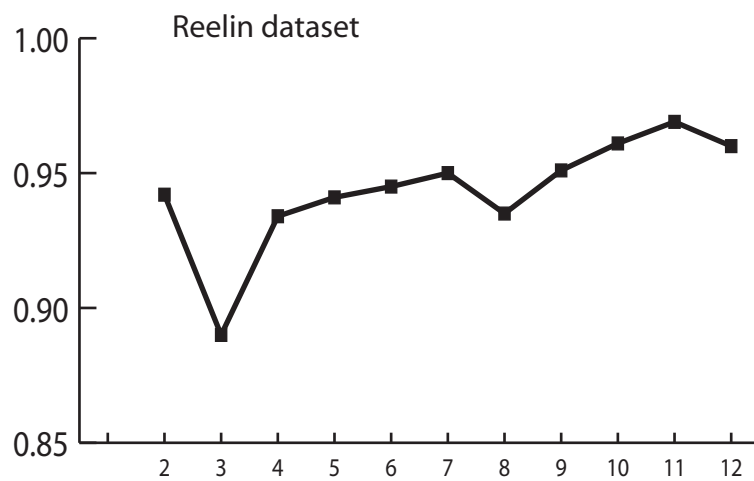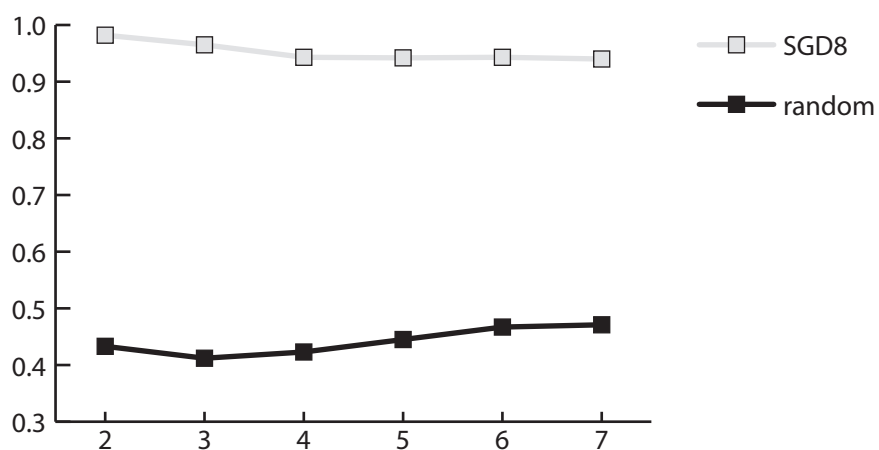

Supplement: Additional File 1 — Cophenetic correlation coefficient. This file contains three graphs showing the cophenetic correlation coefficient for the SGD8, Reelin and random datasets. [file 1471-2105-7-41-S1.PDF]
