# Supplementary material for: Discovering semantic features in the literature: a foundation for building functional associations
Source: BMC Bioinformatics. 2006 Jan 26;7:41. doi: 10.1186/1471-2105-7-41 (PMC1386711; doi:10.1186/1471-2105-7-41)
Supplement: Additional File 4 — Reelin dataset analysis by SVD and NMF. Descriptive comparison of the results obtained by clustering of gene profiles obtained by SVD and NMF algorithms. Includes hierarchical trees. [file 1471-2105-7-41-S4.pdf]

Analysis of the ‘Reelin dataset’: a comparison of SVD and NMF-based profile clustering

The ‘Reelin dataset’ contains 50 genes related to cancer biology, Alzheimer’s disease and development, including 5 genes that are involved in the Reelin pathway. It was proposed and analyzed by Homayouni *et al.* (2005).

As the exact set of document used by Homayouni *et al*. is not readily available, we have analyzed the 50 gene set provided by them, using the literature relevant to the human and mouse genes as obtained from Entrez Gene [31], following the procedure described by Homayouni *et al.* (2005). We provide this literature corpus as supplementary data in the project web page to support future comparative studies.

From a literature corpus of 4,378 distinct articles, a matrix of 50 genes and 1,865 terms was obtained (terms were included if found in at least 10 and no more than 40 genes). See ‘Methods’ section for further details.

NMF-based literature profiles for the gene set were obtained by 10 independent NMF factorizations (k=7) of the gene-term frequency matrix. Profiles were normalized by z-score to make independent factorizations comparable.

Similarly, SVD-based literature profiles were obtained by SVD factorization of the same gene-term frequency matrix. Profiles were truncated so as to contain the first 25 factors (accounting for the 99.9% of the variability in the dataset).

Pair-wise gene distances were calculated from both profile sets as one minus the cosine of the included angle between term vectors. Hierarchical clustering was performed following the procedure described by Homayouni *et al.* (2005), using PHYLIP version 3.65 and the Fitch-Margoliash method.

Overall clustering provided similar local gene-relationships, although differed in the localization a number of genes within general clusters (genes are highlighted with numbered arrows in the hierarchical trees):

1. *FYN* (cancer) is one of the genes described to be involved in the Reelin pathway. It is associated in a group of cancer genes by SVD, while related to the association of the main cancer and development/cancer groups in NMF tree.
2. *SCH1* (cancer) is among a group of Alzheimer genes in SVD tree, while is placed in a cancer group in NMF tree.
3. *TGFB1* (development/cancer) is connected to *CDK5* (development/Alzheimer) and HAPT (alzheimer) subgroup in SVD tree, while associated to a cancer group in NMF tree.
4. *KIT* (cancer) is associated to development/cancer genes in SVD tree, while it is in the main cancer group in NMF tree.
5. *ETS1* (cancer) is associated with *NRAS* (cancer) and *PAX3* (development/cancer) in SVD tree, while grouped with other cancer genes in NMF tree.
6. *NRAS* (cancer) is associated with *ETS1* (cancer) and PAX3 (development/cancer) in SVD tree, while grouped with other cancer genes in NMF tree.

7. *DNHT1* (cancer) is associated with a group of development/cancer genes in SVD tree. It is placed in the cancer group in NMF tree.
8. *APOE* (Alzheimer) is associated to the superset of the main cancer/development, Alzheimer and cancer groups in SVD tree, while closely related to *LRP1* (Alzheimer) in NMF tree.

Subclusters found in both methods, although found within different clusters:

1. CDK5, CDK5R, CDK5R2, (development/alzheimer) MAPT (alzheimer) is close to development/cancer group in SVD tree while close to Alzheimer group in NMF tree.

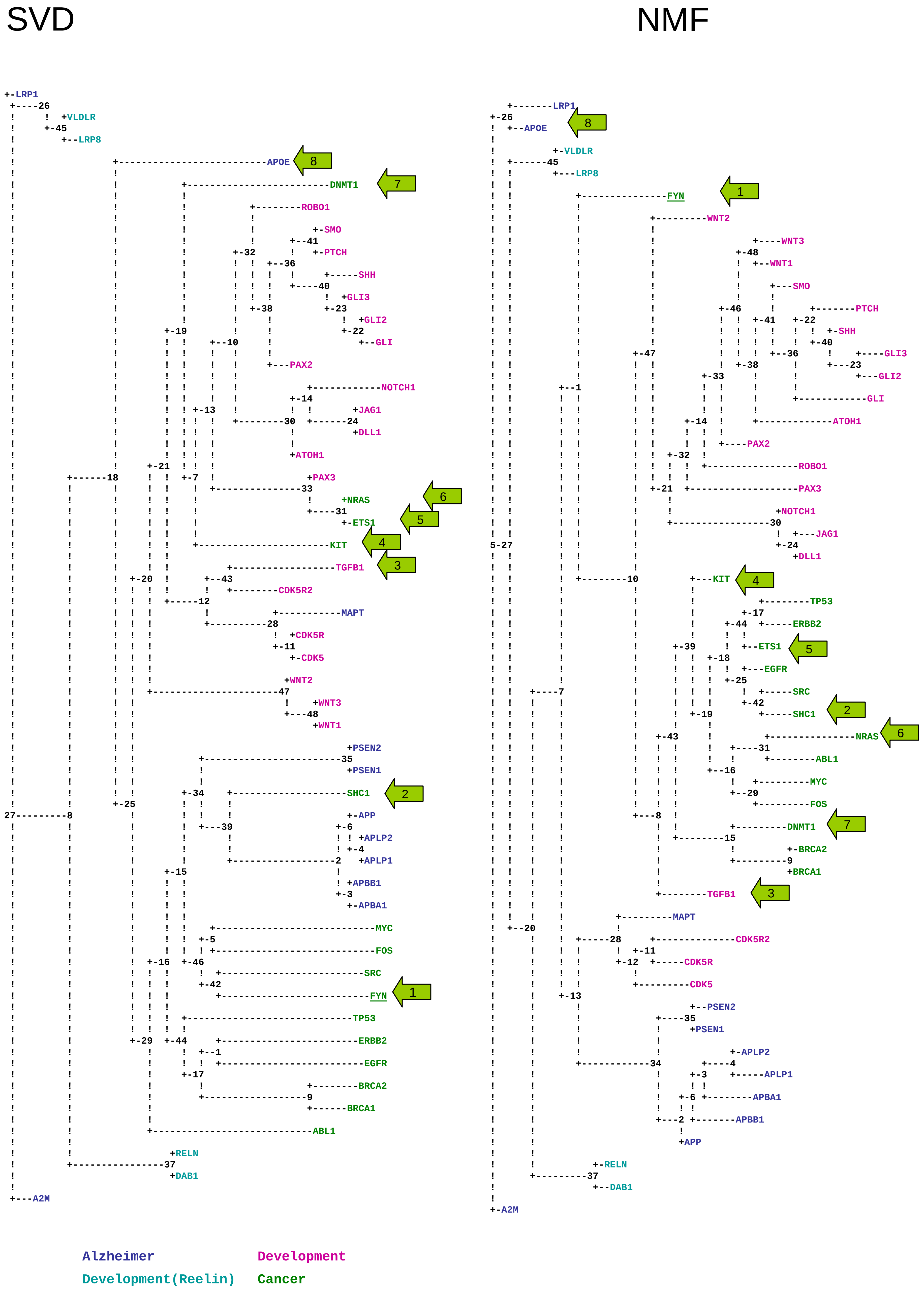

SVD
NMF
LRP1
VLDLR
LRP8
APOE
DNMT1
ROBO1
SMO
PTCH
SHH
GLI3
GLI2
GLI
PAX2
NOTCH1
JAG1
DLL1
ATOH1
PAX3
NRAS
ETS1
KIT
TGFB1
CDK5R2
MAPT
CDK5R
CDK5
WNT2
WNT3
WNT1
PSEN2
PSEN1
SHC1
APP
APLP2
APLP1
APBB1
APBA1
MYC
FOS
SRC
FYN
TP53
ERBB2
EGFR
BRCA2
BRCA1
ABL1
RELN
DAB1
A2M
Alzheimer
Development
Development(Reelin)
Cancer
