# Supplementary material for: Discovering semantic features in the literature: a foundation for building functional associations
Source: BMC Bioinformatics. 2006 Jan 26;7:41. doi: 10.1186/1471-2105-7-41 (PMC1386711; doi:10.1186/1471-2105-7-41)
Supplement: Additional File 3 — Example representation of literature profiles. This file contains the representation of a literature profile for gene PET54 as obtained in the vector space model, a clustered space and the semantic space. [file 1471-2105-7-41-S3.pdf]

PET54 literature profiles

Literature relevant to a given gene might discuss several functional roles of the gene in the cell. To illustrate the differences in terms of literature profiles we compare the representation of genes using the vector space model, the clustered space model, and our own method (semantic profile). We provide the representations of a particular gene (*PET54*) from the SGD8 dataset analyzed in the manuscript, as well as the stemmed term sets that can be used to interpret each representation.

PET54 encodes a protein, located in the mitochondrial inner membrane, required for splicing the COX1 intron AI5 beta and translation of the COX3 mRNA.

1. **PET54 in vector space representation (ordered by decreasing weights).**

This representation is obtained from the gene-term frequency matrix calculated as described in the Methods section (i.e. it is the same gene-term frequency matrix used in NMF analysis)

Dimension: 2365. Only nonzero weight terms are shown.

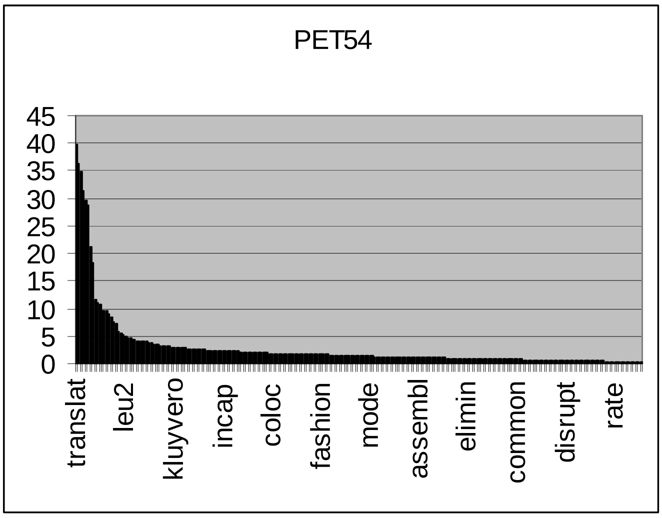

Gene profiles in the vector space model can be analyzed by the examination of most relevant terms (ordered by decreasing weights). E.g. top10 terms of *PET54* reveal important hints of the biological characterization of this gene.

translat
oxidas
mitochondri
cytochrom
leader
intron
mrna
beta
excis
splice

The vector space representation of this gene effectively accounts for several of its characteristics, as shown by the stemmed terms with highest weights (***translat***, *oxidas*, ***mitochondri***, *cytochrom*, *leader*, ***intron***, ***mrna***, *beta*, *excis*, *splice*). However, the vector used for this representation has a very large dimension (dim=2,365).

2. *PET54* in clustered-based representation (ordered by decreasing weights).

In order to obtain an equivalent clustered space model using the vector space representation, we clustered the SGD8 dataset using k-means algorithm (employing the same gene-term frequency matrix used in NMF analysis). In a similar evaluation to that performed on the semantic features, we provided the top 10 terms of the centroids (ordered by decreasing weighting) of each of the 11 clusters to the four experts for interpretation. They were able to understand 8 of the 11 clusters. From the 3 remaining, only one of the experts labeled 2 clusters, while one cluster was not interpretable by none any of the experts.

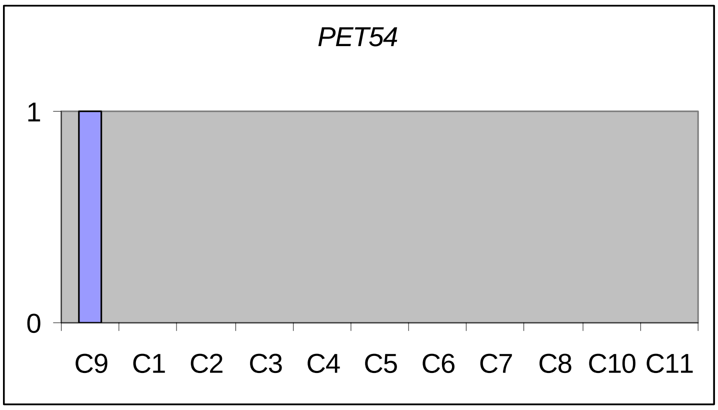

Gene profiles in this space can be analyzed by the examination of most relevant terms of the corresponding cluster. E.g. the PET54 profile can interpreted by the top10 terms of the centroid in cluster #9.

Table: Top10 stemmed terms corresponding to the centroids of the 11 clusters obtained by k-means algorithm. Labels shown if term set was meaningful to at least two of the four experts. Relevant feature for PET54 is highlighted in red.

Clus1 *No label*	Clus2 *Chromatin remodeling*	Clus3 *Transport*	Clus4 *Lipid metabolism*	Clus5 *Mitosis*	Clus6 *No label*
synthetas	nucleosom	v-atpas	sterol	spindl	ribosom
fatti	swi	vacuolar	sphingolipid	kinetochor	actin
trna	histon	golgi	ergosterol	cyclin	translat
peroxisom	snf	vesicl	inositol	centromer	pheromon
acet	chromatin	vacuol	ceramid	checkpoint	gcn4
ligas	remodel	copper	phospholipid	microtubul	alpha
coli	tbp	transport	reductas	anaphas	mate
oxygen	acetyl	membran	phosphatidylcholin	mitosi	kinas
enzym	h2a	iron	phosphatidylserin	spb	wall
ester	rsc	snare	cytochrom	mitot	calcineurin

Clus7 *No label*	Clus8 *Transport*	Clus9 *Mitochondria*	Clus10 *DNA replication*	Clus11 *DNA repair*
glucos	autophagi	mitochondri	replic	repair
permeas	copii	inner	dna	ner
transport	vesicl	preprotein	telomer	tfiih
hexos	autophagosom	export	repair	excis
nitrogen	conjug	mrna	orc	dna
uptak	coat	mitochondria	dsb	mismatch
camp	cargo	transloc	pcna	rad6
snf1	vacuol	hsp70	rfc	damag
trehalos	membran	outer	checkpoint	apn1
ubiquitin	golgi	membran	mcm	rad1

3. PET54 in NMF representation (ordered by decreasing weights).

Dimension: 8.

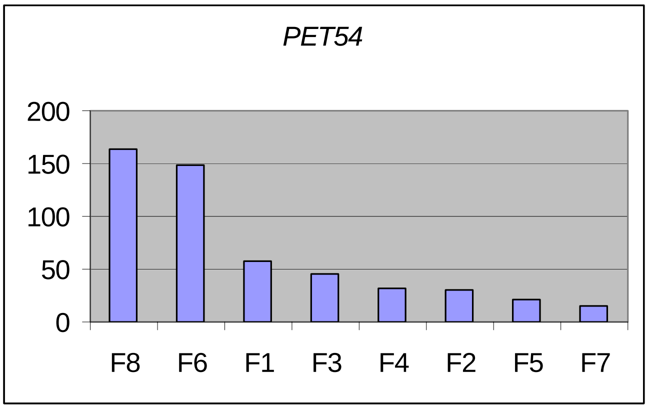

NMF representation was obtained as described in the manuscript.

The representation of *PET54* by means of NMF involves the linear combination of 8 features. Gene profiles in this space can be analyzed by the examination of most relevant terms in each feature. This profile shows highly significant weights to both the *mitochondria* and the *protein synthesis* features, revealing a more complete description of the gene in terms of local features. Although this particular gene was finally assigned to cluster G (*mitochondria*), similar to the cluster created by k-means, its literature profile provides a more detailed representation using a reduced set of features.

Table: Top 10 stemmed terms in the k=8 semantic features obtained for a NMF experiment (ordered by decreasing importance). Labels show topical interpretations provided by experts (including more concrete topics in parenthesis). Relevant features for PET54 are highlighted in red

F1 DNA metabolism (DNA replication)	F2 DNA metabolism (DNA repair)	F3 Metabolism / Stress / Degradation	F4 Transciption (chromatin)	F5 Cell Division (mitosis)	F6 Miochondria	F7 Transport (vesicular trafficking)	F8 Protein synthesis
Replic<br>pcna<br>dna<br>ner<br>damag<br>checkpoint<br>rfc<br>pol<br>polymeras<br>rad6	repair<br>telomer<br>dsb<br>recombin<br>mismatch<br>dna<br>rad52<br>excis<br>rad51<br>endonucleas	glucos<br>fatti<br>heat<br>stress<br>endoplasm<br>reticulum<br>proteasom<br>phosphatas<br>atpas<br>sphingolipid	actin<br>swi<br>nucleosom<br>snf<br>histon<br>chromatin<br>elong<br>mate<br>silenc<br>polar	spindl<br>cyclin<br>kinetochor<br>hsp90<br>chaperon<br>scf<br>anaphas<br>mitosi<br>centromer<br>mitot	mitochondri<br>preprotein<br>mitochondria<br>inner<br>transloc<br>outer<br>membran<br>matrix<br>oxid<br>translocas	transport<br>vesicl<br>vacuolar<br>vacuol<br>membran<br>nitrogen<br>secretori<br>autophagi<br>cytoplasm<br>sort	translat<br>mrna<br>trna<br>alpha<br>gcn4<br>beta<br>gtp<br>phosphoryl<br>exchang<br>kinas
